# Regression Reconstruction from a Retrospective Sample

**DOI:** 10.1016/j.ecosta.2020.10.003

**Published:** 2023-01

**Authors:** Christiana Kartsonaki, D. R. Cox

**Affiliations:** a MRC Population Health Research Unit, Nuffield Department of Population Health, University of Oxford, Oxford OX3 7LF, UK; b Nuffield College, Oxford OX1 1NF, UK

**Keywords:** Bias removal, Case-control study, Indirect sampling

## Abstract

The simplest form of retrospective study allows the reconstruction of the dependence between a binary outcome, Y, representing the contrast between cases and controls, and one or more explanatory variables. A different objective for such situations is considered, in which there are distinct explanatory variables, say (W,X) determining Y. Reconstruction of the originating distribution of (W,X) from the case-control data is considered for both continuous and binary variables. Emphasis is on the linear regression coefficient of W on X. That coefficient, but not the relevant intercept, shows considerable stability, as shown by theory and simulations. An approximation to the value of the coefficient not conditioning on Y is given.^1^

## 1 Introduction

One rather general formulation of the challenge of interpreting observational studies is to suppose data available on a sample of individuals with three broad types of observation, outcomes, Y, explanatory variables, W, and background or intrinsic variables, X. The ultimate objective of study is usually the dependence of Y on W, allowing for the presence of X. An experiment or intervention will study this directly, including often an element of randomization of W. Observational studies are constrained in various ways, implying that the distributions generating the data may be only indirectly related to the distribution of interest.

In particular, in a case-control design inclusion in the data depends strongly on an outcome variable. In the present paper we suppose, somewhat unusually, emphasis lies on the dependence among the explanatory variables, in particular that of W on X in the underlying population, marginalizing over Y. Reconstruction of the underlying dependence of interest is not direct and it has been pointed out (Nagelkerke, Moses, Plummer, Brunham, Fish, 1995, Lee, Mc Murphy, Scott, 1997, Jiang, Scott, Wild, 2006, Lin, Zeng, 2009, Wei, Carroll, Müller, Van Keilegom, Chatterjee, 2013, Xing, Mc Carthy, Dupuis, Cupples, Meigs, Lin, Allen, 2016) that some methods in the literature may be misleading.

An area where this issue often arises is genetic epidemiology. Many genome-wide association studies (GWAS) have a case-control design because their main aim is to discover associations of genetic variants with relatively rare outcomes, but often data on other variables are collected and the data are re-used to assess the associations with other variables in the case-control sample. Lin and Zeng (2009) demonstrated that some approaches commonly employed in applications give biased estimates of the association of interest and proposed methods to address this issue. Monsees et al. (2009) discussed the issues with a focus on GWAS and testing. Dai and Zhang (2014) studied the Mendelian randomisation estimator for the relationship of a continuous exposure with an outcome in a case-control study.

Because the population proportion of cases is in general unknown, corrections for weighted sampling based on unequal but known probabilities of selection (Horvitz and Thompson, 1952) are not available, except possibly as the basis for a sensitivity analysis.

The emphasis in the present note is on the formal relations involved, not on explicit details of estimation procedures.

In section 2 we give the formulation of the problem and theoretical relations involved for linear regression when W is continuous and in particular a formula for the reconstructed regression coefficient. In section 3 we present some results from a simulation study illustrating the theoretical findings. Section 4 introduces the corresponding relationships when all variables are binary, and in section 5 the conclusions are discussed.

## 2 Theory

**Fig. 1:**
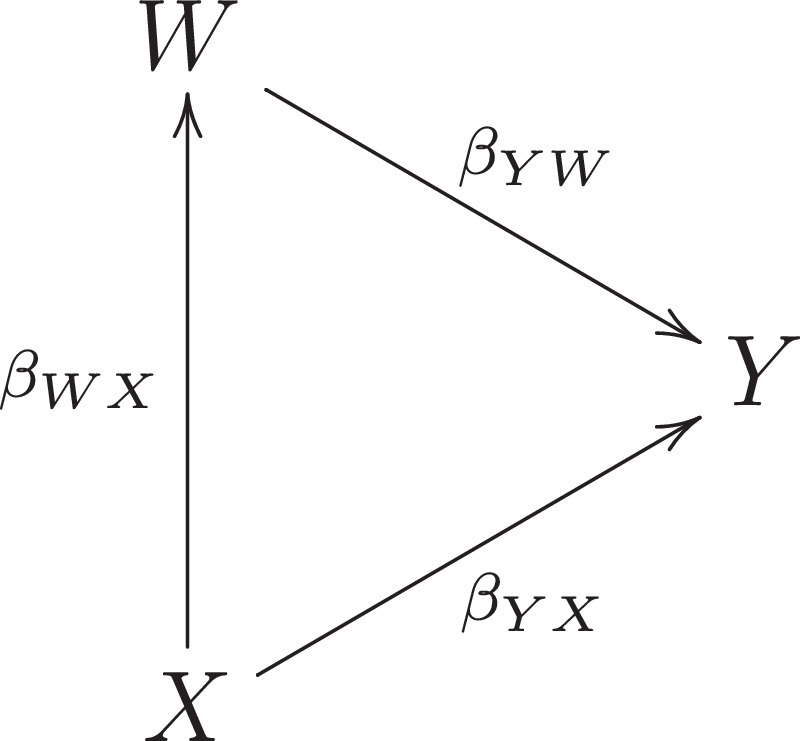
Path diagram.

To study these issues in their simplest form we consider two random variables (X,W) whose population distribution is of interest. The case/control binary outcome Y depends on (X,W) and defines two random samples conditionally respectively on Y=1, cases, and Y=0, controls (Figure 1). From these we wish to reconstruct the population distribution of (X,W). Our arguments are general but we focus on the linear regression coefficient, $\beta_{WX},$ of W on X. We treat both variables as one-dimensional; the results extend directly to vector (W,X).

An instructive but extreme special case arises when Y is conditionally independent of W given X. Then also W is conditionally independent of Y given X so that the form of the regression relation of W on X is the same within cases and within controls and within the population. This is concordant with the general notion that in fitting regression relations the explanatory variables are typically regarded as fixed at their observed values. The joint distribution of (W,X) is, however, in general different in cases from that in controls. To estimate the linear least squares regression coefficient of W on X we may, however, in this situation find the regression coefficients and their standard errors separately within cases and within controls and, preferably subject to an informal check of consistency, calculate a weighted mean.

More generally we suppose without loss of generality that E(W)=E(X)=0 and also that

$$P\left(Y=1|W=w,X=x\right)=L\left(\alpha +\beta_{YW.X}w+\beta_{YX.W}x\right),$$

where L(.) is an increasing function with values in (0, 1). The regression coefficients, such as $\beta_{YW.X},$ are defined for a given function L(.), so that if different such functions are involved in a specific study an extended notation would be required. Natural choices for L(.) are the standardized normal integral and the logistic function. Another important possibility, normally useful, however, only over a restricted range, is the linear in probability model, L(x)=x, for 0≤x≤1. It is known that if the data are concentrated in the range of probabilities say in (0.2,0.8) empirical choice between different ‘dose-response’ relations such as logistic, integrated normal and linear is feasible only with very large amounts of data (Chambers and Cox, 1967). Then marginally in the population, P(Y=1)=α. For the cases, Y=1, we have

$$\begin{array}{ccc} f_{WX|Y}\left(w,x;1\right) & = & f_{WX}\left(w,x\right)\left(\alpha +\beta_{YX.W}x+\beta_{YW.X}w\right)/\alpha \\ \text{and}\\ f_{X|Y}\left(x;1\right) & = & f_{X}\left(x\right)\left(\alpha +\beta_{YX}x\right)/\alpha , \end{array}$$

on using the relation that $\beta_{YX}=\beta_{YX.W}+\beta_{YW.X}\beta_{WX}.$ It follows that the conditional distribution of W given X=x within the cases, Y=1, is

(1) $$f_{W|X,Y}\left(w;x,1\right)=f_{W|X}\left(w;x\right)\frac{\alpha +\beta_{YX.W}x+\beta_{YW.X}w}{\alpha +\beta_{YX}x}.$$

To obtain results for Y=0, the controls, we replace α by 1−α and reverse the sign of the regression coefficients $\left(\beta_{YX.W},\beta_{YW.X},\beta_{YX}\right)$.

Thus the conditional distribution of W given X is the same in cases and controls and in the population if and only if $\beta_{YW.X}=0,$ consistently with the more general result noted previously. However the conditional mean of W in (1) is, on writing for the population $E\left(W|X=x\right)=\beta_{WX}x$ and simplifying,

$$E\left(W|X=x,Y=1\right)=\beta_{WX}x+\frac{\beta_{YW.X}\sigma_{W.X}^{2}}{\alpha +\beta_{YX}x},$$

where $\sigma_{W.X}^{2}$ is the conditional variance of W around its least squares regression on X.

Thus when all regression coefficients are positive the regression line of W on X among the cases is somewhat lower than its population form but has the same slope. The replacement of α by 1−α for the controls implies that because α is typically small the distortion among the controls is much smaller.

In many applications, however, especially where some probabilities are quite small, the linear in probability model will not be reasonable. Indeed the most common reason for use of a case-control design is that cases are rare in the population, indeed possibly very rare. In such situations the relation between X and W in the population will be close to that in the controls and the linearity of the assumed dependence of P(Y=1∣X=x,W=w) suspect. We give a more realistic formulation later.

A more detailed analysis of the linear in probability model shows that if the regression of W on X were studied directly ignoring case/control status then to a first approximation the slope would be unchanged but the position of the line displaced.

For a more refined analysis abandoning the linearity assumption, we assume (X,W) to have a bivariate normal distribution, taken without loss of generality to have zero means. The regression coefficient of W on X is again denoted by $\beta_{WX}$. We assume further that instead of (1)

$$P\left(Y=1|W=w,X=x\right)=\Phi \left(-\alpha +\beta_{YW.X}w+\beta_{YX.W}x\right),$$

where Φ(.) is the standard normal cumulative distribution function. This leads, after integrating over the conditional distribution of W given X=x, and then over the distribution of X, to

$$\begin{array}{c} P\left(Y=1|X=x\right)=\Phi \{\left(-\alpha +\beta_{YX}x\right)/\tau \}\\ \text{and}\\ P(Y=1)=\Phi (-\alpha /\gamma ). \end{array}$$

Here $\tau^{2}=1+\beta_{YW.X}^{2}\sigma_{W.X}^{2},\;\;\;\gamma^{2}=1+\tau^{2}+\beta_{YX}^{2}.$ It follows that

$$f_{W|X,Y}\left(w;x,1\right)=f_{W|X}\left(w;x\right)\Phi \left(-\alpha +\beta_{YW.X}w+\beta_{YX.W}x\right)/\Phi \left\{\left(-\alpha +\beta_{YX}x\right)/\tau \right)\},$$

with a complementary expression given Y=0. If we assume that the population regression of W on X is linear with normal errors we may replace the first factor on the right-hand side by $\sigma_{W.X}^{-1}\phi \left(\left(w-\beta_{WX}x\right)/\sigma_{W.X}\right)$. In line with earlier results $f_{W|X,Y}\left(w;x,1\right)=f_{W|X}\left(w;x\right)$ if and only if $\beta_{YW.X}=0$ implying that case/control status is independent of W given X. In general, if we standardize W and X to have zero means and unit variances the regression coefficients involving Y are likely to be numerically small in realistic situations and expansion leads to

$$f_{W|X,Y}\left(w;x,1\right)=f_{W|X}\left(w;x\right)\left\{1+\lambda \left(-\alpha \right)\beta_{YW.X}\left(w-\beta_{WX}x\right)\right\}+O\left(\beta_{Y}^{2}\right),$$

where λ(z)=ϕ(z)/Φ(z), related to Mills ratio. For controls, Y=0, change the sign of $\beta_{YW.X}$ and change α to 1−α.

That is, to this order the impact of case-control sampling on the regression of W on X is to leave the slope unchanged but induce translations of the regression line in opposite directions for cases and controls.

In particular it follows that to this order for the cases

$$E\left(W|X=x,Y=1\right)=\beta_{WX}x+\lambda \left(-\alpha \right)\beta_{YW.X}\sigma_{W.X}^{2}+O\left(\beta_{Y}^{2}\right),$$

where $\beta_{Y}$ in the last term refers to all regression coefficients with Y as outcome variable. For the controls, again reverse the appropriate signs and change α to 1−α.

Inclusion of quadratic terms in the $\beta_{Y}$ shows that relatively complicated nonlinearities are involved. Typically cases are rare, so that α>0 and Φ(−α) is small. There is an upward displacement of the regression line but to this order no change in slope. The downwards shift in the line for controls is by contrast much smaller because now the denominator of λ(α) is close to one whereas the numerator is usually small.

The conditional expectation of W given X=x among the cases now follows on multiplying by w and integrating.

(2) $$E\left(W|X=x,Y=1\right)\approx \beta_{WX}x+\sigma_{W.X}\tau^{-1}\phi \left\{\left(-\alpha +\beta_{YX}x\right)/\tau \right\}/\Phi \left\{\left(-\alpha +\beta_{YX}x\right)/\tau \right\},$$

where ϕ(.) is the standardized normal density. For the controls, reverse the signs of $\alpha ,\beta_{YX}$. The integral is best approximated by the delta method, that is local linearization around the expected value of W, namely $\beta_{WX}x,$ to give the approximations

(3) $$E\left(W|x,1\right)\approx \beta_{WX}x\left\{\Phi \left(-\alpha +\beta_{YX}x\right)+\sigma_{W.X}^{2}\beta_{YW.X}\phi \left(-\alpha +\beta_{YX}x\right)\right\}/\Phi \{\left(-\alpha +\beta_{YX}x\right)/\tau \}.$$

Here ϕ(.) is the standardized normal density. For the controls, Y=0, change the sign of the arguments of Φ(.).

The second term in (2) specifies a nonlinear dependence on x. It is most simply summarized by the slope at x=0 thus changing the linear regression coefficient to

(4) $$\beta_{WX}+\beta_{YX}\sigma_{W.X}\tau^{-1}\phi \left(-\alpha /\tau \right)/\Phi \left(-\alpha /\tau \right).$$

Now for negative z a convenient first approximation is that ϕ(z)/Φ(z)≈−z, in effect the leading term of an asymptotic expansion of Φ(z) for large negative z, underestimating the ratio. This leads to a simple approximation to the regression coefficient among the cases of

(5) $$\beta_{WX}+\beta_{YX}\sigma_{W.X}\alpha /\tau^{2}.$$

For controls, however, a quite different approximation has to be used because Φ(α/τ), being the population proportion of cases, is no longer small. The second term in (4) is thus typically small and the regression coefficient in the controls thus only slightly different from that in the population.

## 3 Some simulations

To illustrate the problem and confirm the results of the previous section we carried out some simulations. We generated a ‘population’ sample with a given relationship between X and W and then selected a case-control sample within that, with case/control status depending on X and/or W. We selected a few plausible values for the relationship between the three variables involved to examine the resulting estimates of the regression of W on X by carrying out various types of analysis: the analysis in the full population and within the case-control sample (incorrectly) conditioning on case/control status, using inverse probability weighting assuming the proportion of cases is known, and applying the proposed correction.

We generated $10^{5}$ values of X∼N(0,1) and $W=\beta_{WX}X+\sqrt{\left(1-\beta_{WX}^{2}\right)}Z,$ where Z∼N(0,1). Case/control status Y was generated to be equal to 1, denoting a case, with probability $L(\alpha +\beta_{YW.X}W+\beta_{YX.W}X),$ where L(·) is the logistic function. Then 2000 cases and 2000 controls were selected at random. For each parameter configuration 250 replicates were generated. We fitted a linear regression of W on X in the full sample, in controls only, in cases only, in both cases and controls adjusting for case/control status, in both cases and controls ignoring case/control status, in both cases and controls with weighting by the inverse of the probability of being selected in the case-control sample and in the case-control sample by using (5). Table 1 is a short summary of a much more extensive study, the summary concentrating on the region where the proportion of cases is relatively small. The most surprising result is the relative stability of the point estimate ${\hat{\beta }}_{WX}$ defined by pooling the data regardless of case/control status. This would not be expected to hold if the relation between W and X was systematically different in cases and controls.

**Table 1:** Simulation results; Continuous X and W; $\beta_{WX}^{\text{pop}}$ is the estimated coefficient from the linear regression of W on X in the population sample, $\beta_{YW.X}$ is the effect of W on Y given X,$\beta_{YX.W}$ is the effect of X on Y given W,${\hat{\beta }}_{WX.0}$ and ${\hat{\beta }}_{WX.1}$ are the estimated coefficients of the regression of W on X in controls and cases only, respectively, ${\hat{\beta }}_{WX.Y}$ is the estimated coefficient from the sample adjusting for Y,${\hat{\beta }}_{WX}$ is the estimated coefficient from the sample ignoring Y,${\hat{\beta }}_{WX}^{\text{IPW}}$ is the estimated coefficient from inverse probability weighted regression, ${\hat{\beta }}_{WX}^{*}$ is the reconstructed estimate using (5) and L(α) is the proportion of cases in the population. Estimates are averages over 250 simulation runs.

				${\hat{\beta }}_{WX}^{\text{pop}}$	${\hat{\beta }}_{WX.0}$	${\hat{\beta }}_{WX.1}$	${\hat{\beta }}_{WX.Y}$	${\hat{\beta }}_{WX}$	${\hat{\beta }}_{WX}^{\text{IPW}}$	${\hat{\beta }}_{WX}^{*}$
$\beta_{WX}$	$\beta_{YW.X}$	$\beta_{YX.W}$	L(α)							
0.5	0.5	0.5	0.02	0.50	0.49	0.49	0.49	0.55	0.50	0.50
			0.1	0.50	0.47	0.46	0.47	0.53	0.49	0.51
		1	0.02	0.50	0.48	0.46	0.47	0.56	0.50	0.50
			0.1	0.50	0.46	0.42	0.44	0.53	0.47	0.53
	1	0.5	0.02	0.50	0.48	0.43	0.45	0.60	0.50	0.51
			0.1	0.50	0.44	0.39	0.41	0.54	0.46	0.54
		1	0.02	0.50	0.46	0.37	0.42	0.59	0.50	0.52
			0.1	0.50	0.41	0.32	0.37	0.54	0.44	0.60
0.8	0.5	0.5	0.02	0.80	0.80	0.79	0.79	0.83	0.80	0.80
			0.1	0.80	0.78	0.78	0.78	0.81	0.79	0.81
		1	0.02	0.80	0.79	0.77	0.78	0.83	0.80	0.80
			0.1	0.80	0.78	0.76	0.77	0.81	0.79	0.82
	1	0.5	0.02	0.80	0.78	0.75	0.77	0.85	0.80	0.81
			0.1	0.80	0.76	0.72	0.74	0.82	0.77	0.83
		1	0.02	0.80	0.78	0.71	0.75	0.84	0.79	0.82
			0.1	0.80	0.75	0.70	0.73	0.82	0.77	0.87

When either $\beta_{YW.X}$ or $\beta_{YX.W}$ is zero and $\beta_{WX}$ is zero, all regressions give the same estimate of $\beta_{WX}$. As $\beta_{YW.X}$ or $\beta_{YX.W}$ increases, the estimates of $\beta_{WX}$ from the regression in cases only, controls only or on both conditioning on case/control status have a downward bias, the bias increasing with increasing $\beta_{YW.X}$ and $\beta_{YX.W}$. The bias is larger when the proportion of cases in the population is larger. The estimates from the weighted regression have a similar pattern but smaller bias. The estimate from the case-control sample obtained without adjusting for case/control status, ${\hat{\beta }}_{WX},$ has a small upward bias when the cases are rare in the population but becomes unbiased when the proportion of cases in the sample becomes closer to the proportion of cases in the population. The reconstructed estimate has a small upward bias.

As a sensitivity analysis the simulation was repeated with X and Z having a $t_{10}$ distribution (Supplementary table). The resulting estimates differed slightly but not substantially from those in Table 1.

## 4 Binary variables

We now describe an argument broadly parallel to that in the previous section for the case where both variables are binary. Consider two binary variables X and W studied at two levels of the variable Y, indicating, again, case/control status, Y. Then in the population for i,j=0,1 we have probabilities

$$p_{ij}^{WX}=\left(1-\pi \right)p_{ij|0}^{WX}+\pi p_{ij|1}^{WX},$$

where $p_{ij}^{WX}$ is the joint probability distribution of W=i,X=j and π=P(Y=1) is the population probability of an individual being a case. Thus any population property, such as, for example, the log odds ratio

$$\psi =\log\{\left(p_{11}^{WX}p_{00}^{WX}\right)/\left(p_{10}^{WX}p_{01}^{WX}\right)\}$$

can be found in terms of quantities that can be estimated and the population parameter π. In particular when π is small, we have that with error $O(\pi^{2})$

$$\begin{array}{ccc} \psi & = & \psi_{0}+\pi (\Delta p_{11}/p_{11|0}+\Delta p_{00}/p_{00|0}\\ & & -\Delta p_{10}/p_{10|0}-\Delta p_{01}/p_{01|0}), \end{array}$$

where $\Delta p_{ij}=p_{ij|1}-p_{ij|0}$ and $\psi_{0}$ is the log odds ratio in the controls, Y=0, and $p_{ij|0}$ is the conditional probability of W=i,X=j in controls. Note that because probabilities sum to one,

$$\Delta p_{11}+\Delta p_{00}-\Delta p_{10}-\Delta p_{01}=0.$$

Thus the adjustment term, the coefficient of π, is particularly important when both $p_{11|1}-p_{11|0}$ and $p_{00|1}-p_{00|0}$ have the same sign.

An alternative approach more closely linked to the results of Section 2 is to regard the binary variables as dichotomized versions of unobserved normally distributed random variables. The earlier results may then be adapted.

## 5 Discussion

We have discussed the relationships involved when fitting a regression on a pair of variables in a non-random sample, selected on the basis of a third variable. We have found that the regression coefficient is relatively stable under different types of analysis and have proposed a correction to reconstruct the coefficient that would have been obtained under no selection. The intercepts from the different analyses vary substantially, as expected.

The simulations show that the regression coefficient estimated conditioning on case/control status has substantial downward bias, whereas the coefficient estimated by ignoring case/control status or using inverse probability weighting are closer to the value estimated from the population from which the case-control sample has been drawn.

The account given here of sampling based on case/control status can be generalized in various ways, the most immediate of which is to replace the scalar explanatory variable X by a vector. When (X,W) are both binary the discussion can be extended to include adjustment for other covariates, including continuous ones. Yet another possibility is to consider the use of instrumental variables to assess the ‘causal’ effect of X on W from case-control sampling. Dai and Zhang (2015) reported that an uncorrected estimate is unbiased in the null case but otherwise biased away from the null.

In the very special case when case/control status, Y, is independent of W given X, there are three sources of information about (W,X), within controls, within cases, and comparison of group means. First approximations to the first two, before adjustment for the special sampling procedure, are given, with their estimated variances, by standard linear regression formulae.

The third estimate, based on the comparison of means is asymptotically independent of the other two and is ${\hat{\beta }}_{B}=\left(\bar{w_{1}}-\bar{w_{0}}\right)/\left(\bar{x_{1}}-\bar{x_{0}}\right)$. Its variance is best found conditionally on the values of X as proportional to the variance of the difference of two independent means. The final estimate is a weighted mean with weights the reciprocals of the estimated variances. A more refined estimate of precision, allowing for errors in the estimated weights, has not been investigated.

The investigation outlined here is one facet of the broad challenge of the study of dependences that can be investigated only indirectly.

## Appendix A Supplementary materials

Supplementary Data S1 Supplementary Raw Research Data. This is open data under the CC BY license http://creativecommons.org/licenses/by/4.0/ Supplementary Data S1
