# Supplementary material for: Regression Reconstruction from a Retrospective Sample
Source: Econom Stat. 2023 Jan;25:87–92. doi: 10.1016/j.ecosta.2020.10.003 (PMC9872473; doi:10.1016/j.ecosta.2020.10.003)
Supplement: Supplementary Data S1 — Supplementary Raw Research Data. This is open data under the CC BY license http://creativecommons.org/licenses/by/4.0/ [file mmc1.docx]

Regression reconstruction from a retrospective sample

Supplementary material

**Simulation code for table 1**

logit <- function(x) (log(x / (1 - x)))

L <- function(x) {
 exp(x) / (1 + exp(x))
 }

beta_YW_X_values <- c(0.5, 1)
beta_YX_W_values <- c(0.5, 1)
beta_WX_values <- c(0.5, 0.8)

intercept_values <- c(logit(0.02), logit(0.1))

n_values <- c(100000)

# sample size of cases and controls as a proportion of total n (has to be <= prop_cases)
prop_0 <- prop_1 <- c(0.02)
# prop_cases: proportion of cases in the population
prop_cases <- c(0.05)

parameters <- expand.grid("beta_YW_X" = beta_YW_X_values, "beta_WX" = beta_WX_values, "beta_YX_W" = beta_YX_W_values, "intercept" = intercept_values, "n" = n_values, "prop_0" = prop_0, "prop_1" = prop_1, "prop_cases" = prop_cases)
parameters$n0 <- parameters$n * parameters$prop_0
parameters$n1 <- parameters$n * parameters$prop_1

sim_continuous <- function(beta_YW_X, beta_WX, beta_YX_W, intercept, n, n0, n1, prop_cases) {

 # n: population size
 # n0: sample size of controls
 # n1: sample size of cases

 set.seed(5)

 coef_all <- list()
 int_all <- list()
 coef_controls <- list()
 int_controls <- list()
 coef_cases <- list()
 int_cases <- list()
 coef_adj <- list()
 int_adj <- list()
 coef_unadj <- list()
 int_unadj <- list()
 coef_ipw <- list()
 int_ipw <- list()
 beta_reconstructed <- list()
 beta_weighted <- list()

 repetitions <- 250

 for (r in 1:repetitions) {
 z1 <- rnorm(n = n, mean = 0, sd = 1)
 z2 <- rnorm(n = n, mean = 0, sd = 1)
 z3 <- rnorm(n = n, mean = 0, sd = 1)

 x <- z1
 w <- beta_WX * z1 + sqrt(1 - beta_WX^2) * z2

 k <- 0.607
 y <- rep(NA, length(x))
 for (i in 1:length(y)) {
 y[i] <- rbinom(n = 1, size = 1, prob = L(intercept + beta_YX_W * x[i] + beta_YW_X * w[i]))
 }

 controls <- sample(x = which(y == 0), size = n0)
 cases <- sample(x = which(y == 1), size = n1)

 # Linear model of $W$ on $x$ on all simulated data:
 coef_all[[r]] <- summary(lm(w ~ x))$coef[2, 1]
 int_all[[r]] <- summary(lm(w ~ x))$coef[1, 1]

 # Linear model of $W$ on $x$ on controls:
 coef_controls[[r]] <- summary(lm(w[controls] ~ x[controls]))$coef[2, 1]
 int_controls[[r]] <- summary(lm(w[controls] ~ x[controls]))$coef[1, 1]

 # Linear model of $W$ on $x$ on cases:
 coef_cases[[r]] <- summary(lm(w[cases] ~ x[cases]))$coef[2, 1]
 int_cases[[r]] <- summary(lm(w[cases] ~ x[cases]))$coef[1, 1]

 # Linear model of $W$ on $x$ on cases and controls, adjusting for case/control status:
 coef_adj[[r]] <- summary(lm(c(w[controls], w[cases]) ~ c(x[controls], x[cases]) + c(rep(0, n0), rep(1, n1))))$coef[2, 1]
 int_adj[[r]] <- summary(lm(c(w[controls], w[cases]) ~ c(x[controls], x[cases]) + c(rep(0, n0), rep(1, n1))))$coef[1, 1]

 # Linear model of $W$ on $x$ on cases and controls, ignoring case/control status:
 coef_unadj[[r]] <- summary(lm(c(w[controls], w[cases]) ~ c(x[controls], x[cases])))$coef[2, 1]
 int_unadj[[r]] <- summary(lm(c(w[controls], w[cases]) ~ c(x[controls], x[cases])))$coef[1, 1]

 # Linear model of $W$ on $x$ on cases and controls, with inverse probability weighting:
 coef_ipw[[r]] <- summary(lm(c(w[controls], w[cases]) ~ c(x[controls], x[cases]), weights = c(rep(((n - (n * prop_cases)) / length(controls)), times = length(controls)), rep(((n * prop_cases) / length(cases)), times = length(cases)))))$coef[2, 1]
 int_ipw[[r]] <- summary(lm(c(w[controls], w[cases]) ~ c(x[controls], x[cases]), weights = c(rep(((n - (n * prop_cases)) / length(controls)), times = length(controls)), rep(((n * prop_cases) / length(cases)), times = length(cases)))))$coef[1, 1]

 # p1: proportion of cases in the population (assumed small)
 theta <- length(which(y == 1))/n

 # new reconstructed estimate of beta
 beta_YX <- summary(glm(y ~ x, family = "binomial"))$coef[2, 1]
 beta_reconstructed[[r]] <- as.numeric(coef_controls[[r]]) + theta * beta_YW_X * beta_YX * var(residuals(lm(w ~ x)))

 # weighted beta
 v0 <- summary(lm(w[controls] ~ x[controls]))$coef[2, 2]^2
 v1 <- summary(lm(w[cases] ~ x[cases]))$coef[2, 2]^2
 beta_weighted[[r]] <- ((as.numeric(coef_controls[[r]]) / v0) + (as.numeric(coef_cases[[r]]) / v1)) / ((1 / v0) + (1 / v1))
 }

 coef_all_mean <- mean(as.numeric(coef_all))
 coef_all_se <- sd(as.numeric(coef_all)) / sqrt(repetitions)

 int_all_mean <- mean(as.numeric(int_all))

 coef_controls_mean <- mean(as.numeric(coef_controls))
 coef_controls_se <- sd(as.numeric(coef_controls)) / sqrt(repetitions)

 int_controls_mean <- mean(as.numeric(int_controls))

 coef_cases_mean <- mean(as.numeric(coef_cases))
 coef_cases_se <- sd(as.numeric(coef_cases)) / sqrt(repetitions)

 int_cases_mean <- mean(as.numeric(int_cases))

 coef_adj_mean <- mean(as.numeric(coef_adj))
 coef_adj_se <- sd(as.numeric(coef_adj)) / sqrt(repetitions)

 int_adj_mean <- mean(as.numeric(int_adj))

 coef_unadj_mean <- mean(as.numeric(coef_unadj))
 coef_unadj_se <- sd(as.numeric(coef_unadj)) / sqrt(repetitions)

 int_unadj_mean <- mean(as.numeric(int_unadj))

 coef_ipw_mean <- mean(as.numeric(coef_ipw))
 coef_ipw_se <- sd(as.numeric(coef_ipw)) / sqrt(repetitions)

 int_ipw_mean <- mean(as.numeric(int_ipw))

 beta_reconstructed_mean <- mean(as.numeric(beta_reconstructed))
 beta_reconstructed_se <- sd(as.numeric(beta_reconstructed)) / sqrt(repetitions)

 beta_weighted_mean <- mean(as.numeric(beta_weighted))
 beta_weighted_se <- sd(as.numeric(beta_weighted)) / sqrt(repetitions)

 return(list("coef_all" = coef_all_mean, "coef_all_se" = coef_all_se, "coef_controls" = coef_controls_mean, "coef_controls_se" = coef_controls_se, "coef_cases" = coef_cases_mean, "coef_cases_se" = coef_cases_se, "coef_adj" = coef_adj_mean, "coef_adj_se" = coef_adj_se, "coef_unadj" = coef_unadj_mean, "coef_unadj_se" = coef_unadj_se, "coef_ipw" = coef_ipw_mean, "coef_ipw_se" = coef_ipw_se, "beta_reconstructed" = beta_reconstructed_mean, "beta_reconstructed_se" = beta_reconstructed_se, "beta_weighted" = beta_weighted_mean, "beta_weighted_se" = beta_weighted_se, "int_all" = int_all_mean, "int_controls" = int_controls_mean, "int_cases" = int_cases_mean, "int_adj" = int_adj_mean, "int_unadj" = int_unadj_mean, "int_ipw" = int_ipw_mean))
 }

sim_continuous_list <- list()
for (i in 1:dim(parameters)[1]) {
 sim_continuous_list[[i]] <- sim_continuous(beta_YW_X = parameters[i,]$beta_YW_X, beta_YX_W = parameters[i,]$beta_YX_W, beta_WX = parameters[i,]$beta_WX, intercept = parameters[i,]$intercept, n = parameters[i,]$n, n0 = parameters[i,]$n0, n1 = parameters[i,]$n1, prop_cases = parameters[i,]$prop_cases)
 }

# results in table
sim_continuous_table <- cbind(parameters, do.call(rbind, lapply(sim_continuous_list, function(x) do.call(cbind, x))))

results_split <- split(sim_continuous_table, paste("n = ", sim_continuous_table$n, ", prop_0 = ", sim_continuous_table$prop_0, ", prop_1 = ", sim_continuous_table$prop_1, sep = ""))

results_table <- data.frame(format(lapply(results_split, function(x) round(ftable(xtabs(cbind(coef_all, coef_controls, coef_cases, coef_adj, coef_unadj, coef_ipw, beta_reconstructed) ~ beta_WX + beta_YW_X + beta_YX_W + I(L(intercept)), x)), 2))[[1]], quote = FALSE))

names(results_table) <- sapply(results_table[1,], as.character)
results_table <- results_table[-1,]
row.names(results_table) <- NULL

kable(results_table)

$\boldsymbol{t}$ distribution

logit <- function(x) (log(x / (1 - x)))

L <- function(x) {
 exp(x) / (1 + exp(x))
 }

beta_YW_X_values <- c(0.5, 1)
beta_YX_W_values <- c(0.5, 1)
beta_WX_values <- c(0.5, 0.8)

intercept_values <- c(logit(0.02), logit(0.1))

n_values <- c(100000)

# sample size of cases and controls as a proportion of total n (has to be <= prop_cases)
prop_0 <- prop_1 <- c(0.02)
# prop_cases: proportion of cases in the population
prop_cases <- c(0.05)

parameters <- expand.grid("beta_YW_X" = beta_YW_X_values, "beta_WX" = beta_WX_values, "beta_YX_W" = beta_YX_W_values, "intercept" = intercept_values, "n" = n_values, "prop_0" = prop_0, "prop_1" = prop_1, "prop_cases" = prop_cases)
parameters$n0 <- parameters$n * parameters$prop_0
parameters$n1 <- parameters$n * parameters$prop_1

sim_continuous <- function(beta_YW_X, beta_WX, beta_YX_W, intercept, n, n0, n1, prop_cases) {

 # n: population size
 # n0: sample size of controls
 # n1: sample size of cases

 set.seed(5)

 coef_all <- list()
 int_all <- list()
 coef_controls <- list()
 int_controls <- list()
 coef_cases <- list()
 int_cases <- list()
 coef_adj <- list()
 int_adj <- list()
 coef_unadj <- list()
 int_unadj <- list()
 coef_ipw <- list()
 int_ipw <- list()
 beta_reconstructed <- list()
 beta_weighted <- list()

 repetitions <- 250

 for (r in 1:repetitions) {
 z1 <- rt(n = n, df = 10)
 z2 <- rt(n = n, df = 10)

 x <- z1
 w <- beta_WX * z1 + sqrt(1 - beta_WX^2) * z2

 k <- 0.607
 y <- rep(NA, length(x))
 for (i in 1:length(y)) {
 y[i] <- rbinom(n = 1, size = 1, prob = L(intercept + beta_YX_W * x[i] + beta_YW_X * w[i]))
 }

 controls <- sample(x = which(y == 0), size = n0)
 cases <- sample(x = which(y == 1), size = n1)

 # Linear model of $W$ on $x$ on all simulated data:
 coef_all[[r]] <- summary(lm(w ~ x))$coef[2, 1]
 int_all[[r]] <- summary(lm(w ~ x))$coef[1, 1]

 # Linear model of $W$ on $x$ on controls:
 coef_controls[[r]] <- summary(lm(w[controls] ~ x[controls]))$coef[2, 1]
 int_controls[[r]] <- summary(lm(w[controls] ~ x[controls]))$coef[1, 1]

 # Linear model of $W$ on $x$ on cases:
 coef_cases[[r]] <- summary(lm(w[cases] ~ x[cases]))$coef[2, 1]
 int_cases[[r]] <- summary(lm(w[cases] ~ x[cases]))$coef[1, 1]

 # Linear model of $W$ on $x$ on cases and controls, adjusting for case/control status:
 coef_adj[[r]] <- summary(lm(c(w[controls], w[cases]) ~ c(x[controls], x[cases]) + c(rep(0, n0), rep(1, n1))))$coef[2, 1]
 int_adj[[r]] <- summary(lm(c(w[controls], w[cases]) ~ c(x[controls], x[cases]) + c(rep(0, n0), rep(1, n1))))$coef[1, 1]

 # Linear model of $W$ on $x$ on cases and controls, ignoring case/control status:
 coef_unadj[[r]] <- summary(lm(c(w[controls], w[cases]) ~ c(x[controls], x[cases])))$coef[2, 1]
 int_unadj[[r]] <- summary(lm(c(w[controls], w[cases]) ~ c(x[controls], x[cases])))$coef[1, 1]

 # Linear model of $W$ on $x$ on cases and controls, with inverse probability weighting:
 coef_ipw[[r]] <- summary(lm(c(w[controls], w[cases]) ~ c(x[controls], x[cases]), weights = c(rep(((n - (n * prop_cases)) / length(controls)), times = length(controls)), rep(((n * prop_cases) / length(cases)), times = length(cases)))))$coef[2, 1]
 int_ipw[[r]] <- summary(lm(c(w[controls], w[cases]) ~ c(x[controls], x[cases]), weights = c(rep(((n - (n * prop_cases)) / length(controls)), times = length(controls)), rep(((n * prop_cases) / length(cases)), times = length(cases)))))$coef[1, 1]

 # p1: proportion of cases in the population (assumed small)
 theta <- length(which(y == 1))/n

 # new reconstructed estimate of beta
 beta_YX <- summary(glm(y ~ x, family = "binomial"))$coef[2, 1]
 beta_reconstructed[[r]] <- as.numeric(coef_controls[[r]]) + theta * beta_YW_X * beta_YX * var(residuals(lm(w ~ x)))

 # weighted beta
 v0 <- summary(lm(w[controls] ~ x[controls]))$coef[2, 2]^2
 v1 <- summary(lm(w[cases] ~ x[cases]))$coef[2, 2]^2
 beta_weighted[[r]] <- ((as.numeric(coef_controls[[r]]) / v0) + (as.numeric(coef_cases[[r]]) / v1)) / ((1 / v0) + (1 / v1))
 }

 coef_all_mean <- mean(as.numeric(coef_all))
 coef_all_se <- sd(as.numeric(coef_all)) / sqrt(repetitions)

 int_all_mean <- mean(as.numeric(int_all))

 coef_controls_mean <- mean(as.numeric(coef_controls))
 coef_controls_se <- sd(as.numeric(coef_controls)) / sqrt(repetitions)

 int_controls_mean <- mean(as.numeric(int_controls))

 coef_cases_mean <- mean(as.numeric(coef_cases))
 coef_cases_se <- sd(as.numeric(coef_cases)) / sqrt(repetitions)

 int_cases_mean <- mean(as.numeric(int_cases))

 coef_adj_mean <- mean(as.numeric(coef_adj))
 coef_adj_se <- sd(as.numeric(coef_adj)) / sqrt(repetitions)

 int_adj_mean <- mean(as.numeric(int_adj))

 coef_unadj_mean <- mean(as.numeric(coef_unadj))
 coef_unadj_se <- sd(as.numeric(coef_unadj)) / sqrt(repetitions)

 int_unadj_mean <- mean(as.numeric(int_unadj))

 coef_ipw_mean <- mean(as.numeric(coef_ipw))
 coef_ipw_se <- sd(as.numeric(coef_ipw)) / sqrt(repetitions)

 int_ipw_mean <- mean(as.numeric(int_ipw))

 beta_reconstructed_mean <- mean(as.numeric(beta_reconstructed))
 beta_reconstructed_se <- sd(as.numeric(beta_reconstructed)) / sqrt(repetitions)

 beta_weighted_mean <- mean(as.numeric(beta_weighted))
 beta_weighted_se <- sd(as.numeric(beta_weighted)) / sqrt(repetitions)

 return(list("coef_all" = coef_all_mean, "coef_all_se" = coef_all_se, "coef_controls" = coef_controls_mean, "coef_controls_se" = coef_controls_se, "coef_cases" = coef_cases_mean, "coef_cases_se" = coef_cases_se, "coef_adj" = coef_adj_mean, "coef_adj_se" = coef_adj_se, "coef_unadj" = coef_unadj_mean, "coef_unadj_se" = coef_unadj_se, "coef_ipw" = coef_ipw_mean, "coef_ipw_se" = coef_ipw_se, "beta_reconstructed" = beta_reconstructed_mean, "beta_reconstructed_se" = beta_reconstructed_se, "beta_weighted" = beta_weighted_mean, "beta_weighted_se" = beta_weighted_se, "int_all" = int_all_mean, "int_controls" = int_controls_mean, "int_cases" = int_cases_mean, "int_adj" = int_adj_mean, "int_unadj" = int_unadj_mean, "int_ipw" = int_ipw_mean))
 }

sim_continuous_list <- list()
for (i in 1:dim(parameters)[1]) {
 sim_continuous_list[[i]] <- sim_continuous(beta_YW_X = parameters[i,]$beta_YW_X, beta_YX_W = parameters[i,]$beta_YX_W, beta_WX = parameters[i,]$beta_WX, intercept = parameters[i,]$intercept, n = parameters[i,]$n, n0 = parameters[i,]$n0, n1 = parameters[i,]$n1, prop_cases = parameters[i,]$prop_cases)
 }

# results in table
sim_continuous_table <- cbind(parameters, do.call(rbind, lapply(sim_continuous_list, function(x) do.call(cbind, x))))

results_split <- split(sim_continuous_table, paste("n = ", sim_continuous_table$n, ", prop_0 = ", sim_continuous_table$prop_0, ", prop_1 = ", sim_continuous_table$prop_1, sep = ""))

results_table <- data.frame(format(lapply(results_split, function(x) round(ftable(xtabs(cbind(coef_all, coef_controls, coef_cases, coef_adj, coef_unadj, coef_ipw, beta_reconstructed) ~ beta_WX + beta_YW_X + beta_YX_W + I(L(intercept)), x)), 2))[[1]], quote = FALSE))

names(results_table) <- sapply(results_table[1,], as.character)
results_table <- results_table[-1,]
row.names(results_table) <- NULL

kable(results_table)

|  |  |  |  |  | coef_all | coef_controls | coef_cases | coef_adj | coef_unadj | coef_ipw | beta_reconstructed |
| --- | --- | --- | --- | --- | --- | --- | --- | --- | --- | --- | --- |
| beta_WX | beta_YW_X | beta_YX_W | I(L(intercept)) |  |  |  |  |  |  |  |  |
| 0.5 | 0.5 | 0.5 | 0.02 |  | 0.50 | 0.49 | 0.47 | 0.48 | 0.55 | 0.51 | 0.50 |
|  |  |  | 0.1 |  | 0.50 | 0.47 | 0.44 | 0.45 | 0.52 | 0.48 | 0.51 |
|  |  | 1 | 0.02 |  | 0.50 | 0.48 | 0.42 | 0.45 | 0.54 | 0.50 | 0.51 |
|  |  |  | 0.1 |  | 0.50 | 0.45 | 0.40 | 0.43 | 0.52 | 0.47 | 0.54 |
|  | 1 | 0.5 | 0.02 |  | 0.50 | 0.47 | 0.37 | 0.42 | 0.58 | 0.50 | 0.51 |
|  |  |  | 0.1 |  | 0.50 | 0.43 | 0.35 | 0.39 | 0.53 | 0.46 | 0.56 |
|  |  | 1 | 0.02 |  | 0.50 | 0.45 | 0.30 | 0.37 | 0.56 | 0.49 | 0.54 |
|  |  |  | 0.1 |  | 0.50 | 0.40 | 0.29 | 0.35 | 0.53 | 0.44 | 0.64 |
| 0.8 | 0.5 | 0.5 | 0.02 |  | 0.80 | 0.79 | 0.78 | 0.79 | 0.83 | 0.80 | 0.80 |
|  |  |  | 0.1 |  | 0.80 | 0.78 | 0.77 | 0.77 | 0.81 | 0.79 | 0.81 |
|  |  | 1 | 0.02 |  | 0.80 | 0.79 | 0.76 | 0.77 | 0.82 | 0.80 | 0.80 |
|  |  |  | 0.1 |  | 0.80 | 0.77 | 0.75 | 0.76 | 0.81 | 0.78 | 0.83 |
|  | 1 | 0.5 | 0.02 |  | 0.80 | 0.78 | 0.72 | 0.75 | 0.84 | 0.80 | 0.81 |
|  |  |  | 0.1 |  | 0.80 | 0.75 | 0.70 | 0.73 | 0.82 | 0.77 | 0.85 |
|  |  | 1 | 0.02 |  | 0.80 | 0.77 | 0.68 | 0.73 | 0.83 | 0.79 | 0.83 |
|  |  |  | 0.1 |  | 0.80 | 0.74 | 0.68 | 0.71 | 0.81 | 0.76 | 0.90 |

**Supplementary table. Simulation results based on *t* distribution with 10 degrees of freedom.**
